# An unusual synchronous ileosigmoid and ileoileal knotting: a case report

**DOI:** 10.1186/1752-1947-8-200

**Published:** 2014-06-18

**Authors:** Nikolaos Andromanakos, Dimitrios Filippou, Stamatis Pinis, Alkiviadis Kostakis

**Affiliations:** 1 Department of General Surgery, Athens “Polykliniki” General Hospital, 10A Glafkis str, GR-15232 Halandri, Athens, Greece; 2 Department of General Surgery, Athens “Athinaion” Medical Center, Athens, Greece; 3 Second Department of General Surgery, Piraeus General Hospital “Agios Pandeleimon”, Piraeus, Greece; 4 Second Department of Propedeutic Surgery, Athens University Medical School, Laiko General Hospital, Athens, Greece

**Keywords:** Gangrene, Ileoileal knot, Ileosigmoid knot, Obstruction, Volvulus

## Abstract

**Introduction:**

Ileosigmoid and ileoileal knotting are two rare entities. They usually present as acute abdomen and the diagnosis is established intraoperatively. The treatment is surgical and should be performed as soon as possible to decrease the incidence of perioperative mortality and morbidity.

**Case presentation:**

We report an unusual case of a 26-year-old Argentine man with ischemic necrosis in parts of his small and large intestine due to combined ileosigmoid and ileoileal knotting. He had an ileal loop of ileum concurrently wrapped around the neck of a sigmoid volvulus and other loops of ileum strangulating them, forming two different tangles of tying. This very rare and unusual entity was diagnosed and managed intraoperatively during a diagnostic laparotomy performed on an emergency basis. Both the gangrenous small bowel loops and the affected sigmoid colon area were resected. The continuation of the intestinal tract was restored by primary end-to-end anastomoses. The present case is unusual and to the best of our knowledge no similar cases of simultaneous ileosigmoid and ileoileal knotting have been described in the literature. The postoperative course of our patient was uneventful and he was discharged from the hospital on the 15th postoperative day. One year later he still remains without symptoms from his intestinal tract.

**Conclusion:**

Simultaneous ileosigmoid and ileoileal knotting is a very rare entity that should be diagnosed and treated surgically on an emergency basis to minimize the high postoperative morbidity and mortality.

## Introduction

Intestinal knotting is the obstruction of an intestinal segment with closed loop phenomenon secondary to knotting of the mesentery. Three types of intestinal knotting have been described in the literature. They are characterized by the segments of the large and small intestine that are affected in this rare entity. Intestinal knotting can be ileosigmoid, ileoileal, or include Meckel’s diverticulum or appendix [1]. In ileosigmoid knotting the sigmoid colon forms the axis around which the small bowel loop encircles. In ileoileal knotting one loop of the ileum remains static around which another loop encircles to form the knot [1,2]. The knot causes intestinal obstruction and gangrene which is usually presented as acute abdomen. Most cases have been described in Africa, India, Asia and East Europe, whereas the entity is very rare in the West [3].

We report the unusual case of a patient with synchronous ileosigmoid and ileoileal knotting causing ischemic necrosis in both small and large intestine. The diagnosis was established intraoperatively. Resection of the gangrenous small bowel and sigmoid colon was performed and the intestinal and colon continuation restored by primary end-to-end anastomoses.

## Case presentation

**Figure 1:**
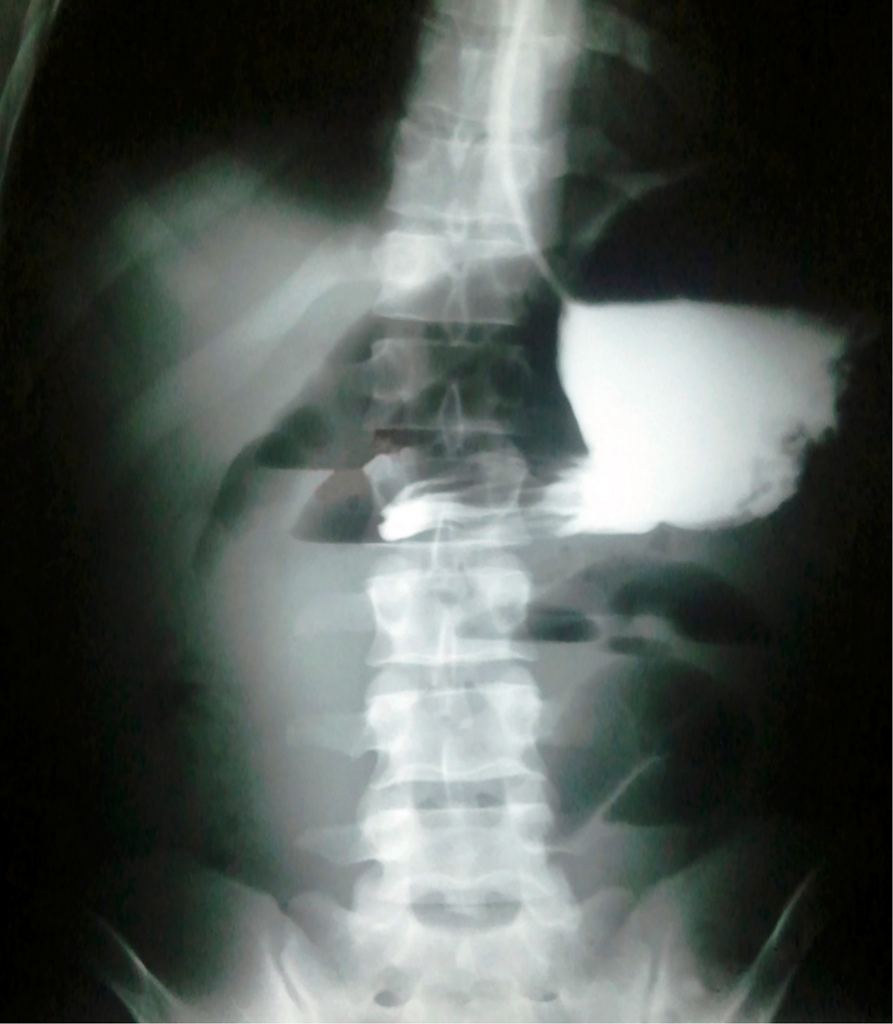
Abdominal X-ray after Gastrografin (sodium diatrizoate and meglumine diatrizoate) administration suggesting obstruction of upper gastrointestinal tract.

**Figure 2:**
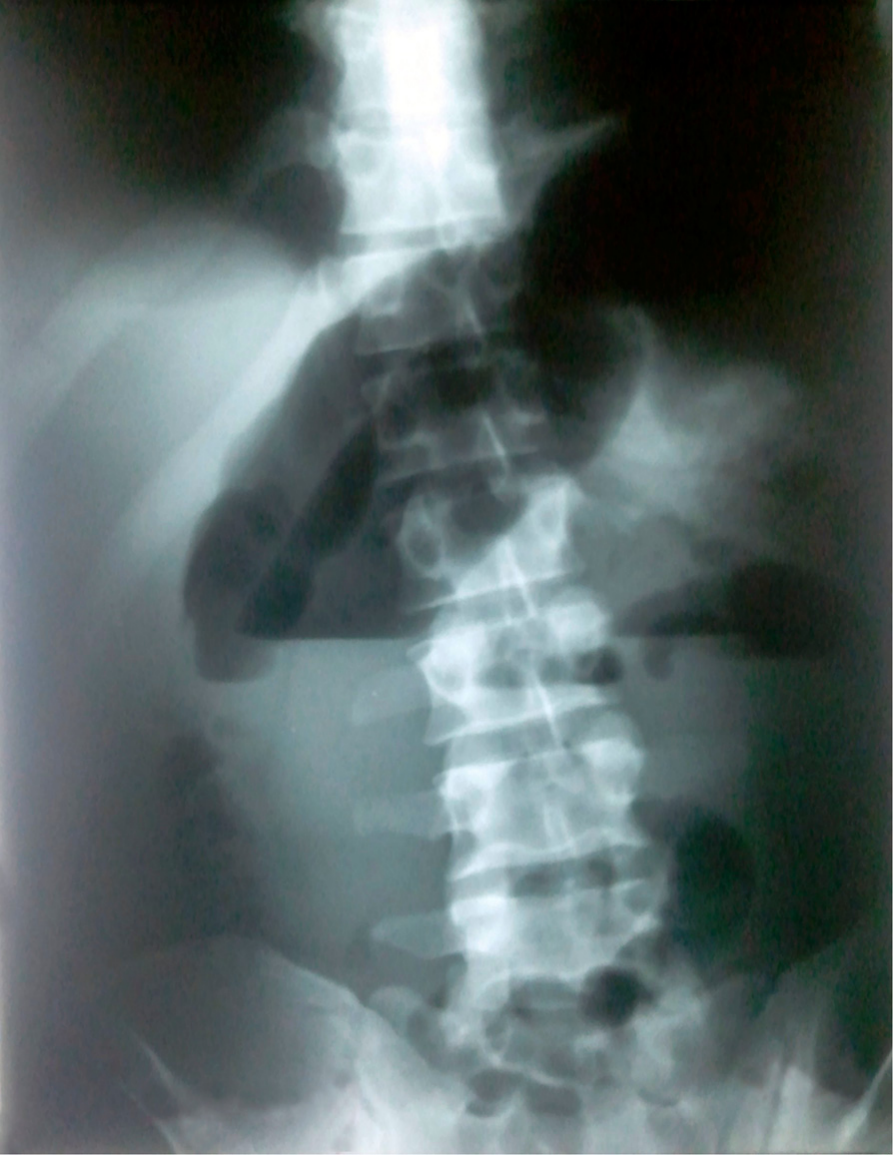
Abdominal X-ray revealing several air-fluid levels of the small bowel and a large air-fluid level of the sigmoid colon.

A 26-year-old Argentine man presented to the emergency department of our hospital complaining of acute severe abdominal pain. The pain started about 6 hours previously and was worsening progressively. A few hours before he visited the hospital he complained of proctalgia and rectal bleeding, and an abdominal discomfort of mild pain, which had started 2 to 4 hours ago; he had been examined by the on-duty physicians who failed to establish an accurate diagnosis. In the physical examination, he seemed to be severely ill and was presenting bradycardia (47 beats per minute) and mild tachypnea, while his body temperature and blood pressure were within the normal values. His abdomen was moderately distended and he presented tenderness with rebound during palpation and rigidity of his abdominal wall. Bowel sounds were absent. In a digital examination, although his rectum was found free of content the pouch of Douglas was very painful. Blood analysis revealed only a mild leucocytosis with neutrophil predominance. His arterial blood gas values were within the normal range. An abdominal radiography showed some rare small air-fluid levels of his small bowel and a large air-fluid level of his sigmoid colon as seen in Figures 1 and 2. An abdominal ultrasound revealed the existence of free fluid around his liver, spleen and Douglas’s pouch. The initial preoperative diagnosis according to existing diagnostic and clinical findings was acute abdomen possibly due to sigmoid volvulus. An urgent diagnostic laparotomy was carried out through a midline incision. The findings were a large amount of bloody fluid and necrosis of an extended area of his small bowel and sigmoid colon. His gangrenous sigmoid colon was distended in torsion surrounded by a loop of ileum strangulating it, forming a tangle of knotting. Furthermore, some gangrenous loops of the ileum were encircled by the same ileal loop, which was wrapped at the basis of the sigmoid volvulus, forming a second tangle of tying. Unraveling these complex intestinal knots was difficult to perform and it was accomplished by using a suction needle to deflate the distended sigmoid colon. The spontaneous untying of the compound intestinal knotting that followed was impressive. Afterwards, the necrotized loops of his ileum (about 150cm) and sigmoid colon were resected. Both small and large intestine continuity were restored by primary end-to-end anastomosis. His postoperative course was uncomplicated and he was discharged on the 15th postoperative day. Since then he remains free of symptoms.

## Discussion

The initial description of intestinal knotting is found on the 16th century by Riverius, while a more detailed description of this rare entity achieved in 1836 by Rokitansky. Ileosigmoid and ileoileal knotting are two very rare entities, particularly in Western countries, that can rapidly evolve to gangrene of the affected bowel segments [1,4]. The predisposing factors of these pathologies may be the coexistence of an elongated, mobile, small bowel mesentery and the redundant sigmoid colon with a narrow basis [2,5-7]. Although these entities are rare many authors suggest that they are more common in young patients, involve ileum loops more often and may be present in pregnant women without history of an abdominal operation. The preoperative diagnosis of these conditions is very difficult or impossible. In most cases the diagnosis is established intraoperatively, as in the present case. However, their clinical presentation as an acute abdominal emergency should not be ignored or accurate management delayed [8]. In addition, the coincidental appearance of acute external hemorrhoids or proctalgia without findings as in the present case, after the onset of abdominal symptoms and a few hours prior to the deterioration and the presentation of the acute abdomen, may announce alterations in the normal function of the colon and may be a sign of distal colonic venous obstruction that should be taken into account more often [9]. The treatment of these rare and unusual conditions is surgical. Any delay of the surgical procedure results in complications which relate to a high mortality rate (0 to 47%) [8,10]. The surgical emergency includes resection of gangrenous bowel parts and primary anastomoses, as we performed in our case, unless not permitted by the intraoperative risk to the patient. In that case most authors suggest resection of the small bowel and primary anastomosis, while in the case of ileosigmoid knotting, Hartmann’s sigmoidectomy, or end colostomy with mucous fistula may be a useful alternative.

## Conclusions

Simultaneous ileosigmoid and ileoileal knotting is a rare entity that should be diagnosed and treated surgically on an emergency basis. An emergency laparotomy as soon as possible and the correct procedure may contribute to a decrease in the high perioperative mortality, which accompanies this terrible pathology. The saving treatment of this rare entity is urgent surgical intervention.

## Patient’s perspective

I would like to remain anonymous.

I write the following to provide assistance to the case report written about my operation. I have no medical knowledge or background. So I write from my own perspective and experience. The problem with my health started as a low diffuse pain in my abdomen and after 2 to 4 hours a quite severe pain appeared in my rectum with a little blood which led me to the hospital “Polykliniki”. I was examined clinically and endoscopically by a gastroenterologist without pathological findings. Afterwards, I came back to my house. Then the abdomen pain was worsening and it forced me to visit again the hospital at the emergency department. The surgeon on-duty, who examined me, diagnosed that I was presenting a severe condition of acute abdomen and then quickly after laboratories and radiological examinations I was led to an operating room. My operation was performed after midnight. The next day I was not feeling as much pain as when I entered hospital. However, I had a tube in my nose and a urocatheter. They were removed after a few days and I slowly started taking drinks and then soups and small meals. The nursing staff attended me with kindness and periodically administered antibiotics, analgesics and serums, according to the doctors’ guidelines. My surgeon, in his morning visit, examined my abdomen, my legs, my chest, my blood pressure and my temperature and then he changed my surgical wound. All the days after my operation were without fever and trouble. I stayed in the hospital 15 days. The stitches of my wound were taken out the day that I was leaving. No problems existed in my recovery and I have been well since.

## Consent

Written informed consent was obtained from our patient for the publication of this case report and any accompanying images. A copy of the written consent is available for review by the Editor-in-Chief of this journal.

## Competing interests

The authors declare that they have no competing interests.

## Authors’ contributions

Our patient was admitted under the care of NA during this episode and was followed up in the outpatient clinic. NA was major contributor in writing the manuscript. DF, SP and AK contributed significantly to the editing of the manuscript and the review of the literature. All authors read and approved the final manuscript.
